# Sex-biased molecular differences in lung adenocarcinoma are ethnic and smoking specific

**DOI:** 10.1186/s12890-023-02387-7

**Published:** 2023-03-24

**Authors:** Xuetao Li, Shuquan Wei, Liaoyuan Deng, HongYan Tao, Mingkai Liu, Ziwen Zhao, Xin Du, Yujun Li, Jun Hou

**Affiliations:** 1 Department of Oncology, Maternal and Child Health Hospital of Hubei Province, Tongji Medical College, Huazhong University of Science and Technology, Wuhan, 430070 China; 2 School of Medicine, South China University of Technology, Guangzhou, 510006 Guangdong China; 3 Department of Pulmonary and Critical Care Medicine, Guangzhou First People’s Hospital, School of Medicine, South China University of Technology, Guangzhou, 510180 Guangdong China; 4 Department of Pulmonary Diseases, The Second Affiliated Hospital of Lanzhou University, Lanzhou, 730000 Gansu China; 5 Department of Hematology, Guangdong Provincial People’s Hospital, Guangdong Academy of Medical Sciences, Guangzhou, 510080 Guangdong China; 6 Center for Medical Research On Innovation and Translation, Institute of Clinical Medicine, Guangzhou First People’s Hospital, School of Medicine, South China University of Technology, Guangzhou, 510180 Guangdong China

**Keywords:** Sex differences, Genomic profiles, Tumor-infiltrating immune cells, Gene expression, Lung adenocarcinoma

## Abstract

**Background:**

Sex-related differences in cancer epidemiology, tumor biology, immune system activity, and pharmacogenomics have been suggested to be important considerations for precision cancer control. Here we elucidated systematically sex biases in genetic variants, gene expression profiles, and immunological landscapes of lung adenocarcinoma patients (LUADs) with different ancestry and smoking status.

**Methods:**

Somatic mutation and mRNA expression data of Asian and Non-Asian LUADs were obtained from public databases. Sex-biased genetic mutations, gene expression, biological pathways, and immune infiltration were identified in the context of smoking status and race.

**Results:**

Among nonsmokers, male-biased mutations were prevalent in Asian LUADs, while few sex-biased mutations were detected in Non-Asian LUADs. *EGFR* was the only mutation whose frequency was significantly higher in females than males in both Asian and Non-Asian nonsmokers. More genes exhibited sex-biased expression in Non-Asian LUADs compared to Asian LUADs. Moreover, genes distinctly expressed in females were mainly related to immune-related pathways, whereas those in males were more involved in activation of DNA repair, E2F_targets, and MYC_targets pathways. We also detected sex-specific immune infiltration in the context of genetic variation. In *EGFR*-mutant LUADs, males had a significantly increased infiltration of CD8 + T cells, whereas resting CD4 + memory T cells were more abundant in females. Additionally, in *KRAS*-mutant LUADs, CD8 + and CD4 + T cells were more abundant in females than males. In addition, we detected all female patients with high *SCGB3A2* expression were exclusively sensitive to immunotherapy, while this phenomenon was not observed in male patients.

**Conclusions:**

Our findings provided evidence that sex-related molecular and cellular components are involved in shaping tumor distinct genetic and immune features, which might have important impact on personalized targeted and immune therapy.

**Supplementary Information:**

The online version contains supplementary material available at 10.1186/s12890-023-02387-7.

## Introduction

Sex differences in susceptibility to cancer have been recognized for a long time. The incidence of cancer is rising more steadily in males than in females, and cancer-related survival in males is also inferior to that in females across multiple cancer types [1]. The higher cancer risk in males is partially explained by hormonal influence or risky behaviors such as smoking and alcohol consumption [2]. Although epidemiologic research has appreciated the hormonal factors and exposure to mutagens as the important reasons for the significant differences between sexes in incidence, severity and outcome of cancers, the biological origins and mechanisms of these differences remain astonishingly unexplored in cancer biology.

Sex differences are also found in response to cancer treatment. Conforti and colleagues demonstrated that male patients with advanced melanoma and non-small cell lung cancer (NSCLC) obtained a significantly better efficacy from anti-CTLA4 or anti-PD-1 monotherapy than female patients [3]. Another study by Conforti showed that compared to male NSCLC patients, female patients acquired significantly more benefit from the anti-PD-1 or anti-PD-L1 combination regimens with chemotherapy [4]. Also, a recent study reported that female patients with metastatic NSCLC and treated with anti-PD-1 inhibitors were more likely to develop immune-related adverse events compared with male counterparts [5]. These observations highlight the importance of considering sex as a variable in clinical research and practice. For instance, we should carefully inspect the potential influence of sex biases in immune infiltration on immune response, including response to cancer immunotherapy.

Recently, scientists have started to correlate the sex differences in clinical behaviors to the architecture and modulation of the cancer genome, as well as functional manifestations of the altered cancer genome. For example, detailed comparisons of genomic profiles between tumors arising in males and females were conducted in recent studies [6, 7]. Impressively, in addition to the differential mutation load observed between sexes, distinct mutation densities and mutation patterns were associated with sex in the same type of tumor; moreover, these sex-specific molecular profiles appeared not uniform across tumor types [6]. A pan-cancer study exploring the impact of sex on molecular profiles showed that male patients with LUAD had a higher frequency of *STK11* mutation than female patients, and *CTNNB1* in hepatocellular carcinoma was more frequently mutated in males than in females [8].

The recognized and proposed determinant factors for sex-related dimorphism in cancer include intrinsic (such as sex hormone and aging) and extrinsic factors (such as tobacco smoke) [9–11]. These factors exert independent or cooperatively direct or indirect regulatory effect on sex-associated cancer biology. Examples include smoke-induced genotoxicity and the pertinent accumulation of genetic variants that similarly demonstrate sex-related disparity. LUAD patients with a long history of exposure to cigarette smoke very often display a high tumor mutation load [12]. On account of the higher smoking prevalence in males than in females, a significantly higher tumor mutational burden has been reported in male patients with NSCLC [13, 14]. Another possible reason for the higher mutation load in males is that the initial accumulation of somatic mutations is approximately a decade earlier in males than in females [9]. Moreover, exposure to first- or second-hand smoke might also contribute to the differences in intratumoral immune infiltrates [15]. In addition, genetic background alone, or more often synergistic with gender, predisposes or regulates genome to specific cancer risk. Substantial evidence suggests that genetic variants seem to differ markedly between Asian and Non-Asian LUADs. For example, the frequency of *EGFR* somatic mutation in Asian LUADs was notably higher than that in western population [16]. However, the sex-biased genetic variants in LUAD from different ancestry remain unclear.

Sex differences in molecular profiles of LUAD patients have yet to be elucidated systematically. Herein we seek to disclose the variations in LUAD behaviors between males and females by exploring the sex-defined heterogeneity in genetic variants, gene expression profiles, and immune profiles in LUAD in regard to smoking status, ethnicity, and mutation-phenotype. And we also seek to explore whether the molecular determinants for immunotherapy response have sex disparities.

## Materials and methods

### Data acquisition

This study made use of data in the public domain. Samples with incomplete clinical information including sex, smoking status, and race were excluded from the analysis. A total of 7 cohorts of high throughput genomic or transcriptomic data were obtained from public data repositories (Additional file 1: Table S1). A cohort from Asian LUADs including somatic mutation data (*n* = 299) and normalized mRNA expression data (*n* = 167) was downloaded from OncoSG (https://src.gisapps.org/OncoSG/) [16]. Somatic mutation data of Chinese LUADs (*n* = 1370) was downloaded from China Pan-cancer (OrigiMed2020) dataset, which was based on deep targeted next-generation sequencing (NGS) of a panel of 450 known cancer-related genes (http://www.cbioportal.org/) [17]. Additionally, normalized mRNA expression data of Chinese LUADs from the CHOICE (*n* = 128) were included in the study [18]. A Non-Asian cohort including somatic mutational data (*n* = 356) and normalized mRNA expression data (*n* = 356) was also downloaded from The Cancer Genome Atlas (TCGA) [19]. Somatic mutation data of Non-Asian LUADs (*n* = 521) was obtained from the Memorial Sloan Kettering Cancer Center (MSK) IMPACT dataset, which was based on MSK-IMPACT sequencing assay with 341-gene or 410-gene MSK-IMPACT panels (http://www.cbioportal.org/) [20]. RNA-seq data of LUADs with immune checkpoint inhibitor (ICB) treatment were obtained from GEO (GSE135222 [21] and GSE166449 [22]). The clinical characteristics of included cases were shown in Additional file 1: Table S2-S3.

### Identification of sex-biased genetic variants

A somatic variant was removed if it met the following criteria: (1) synonymous or UTR variants; (2) the non-silent mutations with < 2% mutation frequency. The MutSigCV (v.1.41) [23] was applied to identify significantly mutated driver genes using a cutoff of false discovery rate (FDR) < 0.05. To rule out the possible effects of race and smoking status on sex-biased genetic variants, datasets were stratified by race and smoking status and analyzed separately. Fisher’s exact test was applied to identify the genetic variants that show significant differences between male and female LUAD patients (FDR < 0.1).

### Analysis of sex-biased gene expression and pathways

We used DESeq2 to identify differentially expressed genes (DEGs) between females and males. The genes with |log2 fold change|> 1 and an adjusted *P* value < 0.05 (Benjamini–Hochberg correction) were considered DEGs. Single-sample gene set enrichment analysis (ssGSEA) was performed to determine enrichment scores for the 50 hallmark gene sets from the Molecular Signatures Database (MSigDB) using R package GSVA [24, 25].

### Analysis of tumor-infiltrating immune cell

We performed current acknowledged algorithms such as CIBERSORTx [26] and xCell [27] to investigate the tumor-infiltrating immune cell landscape of LUAD samples. The CIBERSORT is a deconvolution algorithm to estimate the proportions of 22 different immune cell types in the sample using bulk transcriptomic data. xCell is a gene signature-based method, which employs a compensation technique to reduce spill-over effects between closely related cell types.

### Statistical analysis

The bias of the clinical characteristics between male and female patients were assessed using Chi-squared test for categorical variables. Fisher’s exact test was used to compare sex differences in genetic variants. Mann–Whitney U test was used for comparison of sex differences in tumor-infiltrating immune cells. Statistical analyses were performed using R (version 3.6.3, https://www.r-project.org/) and SPSS (version 26.0).

## Results

### Sex-biased genomic profiles in LUADs differed by race and smoking status

**Fig. 1:**
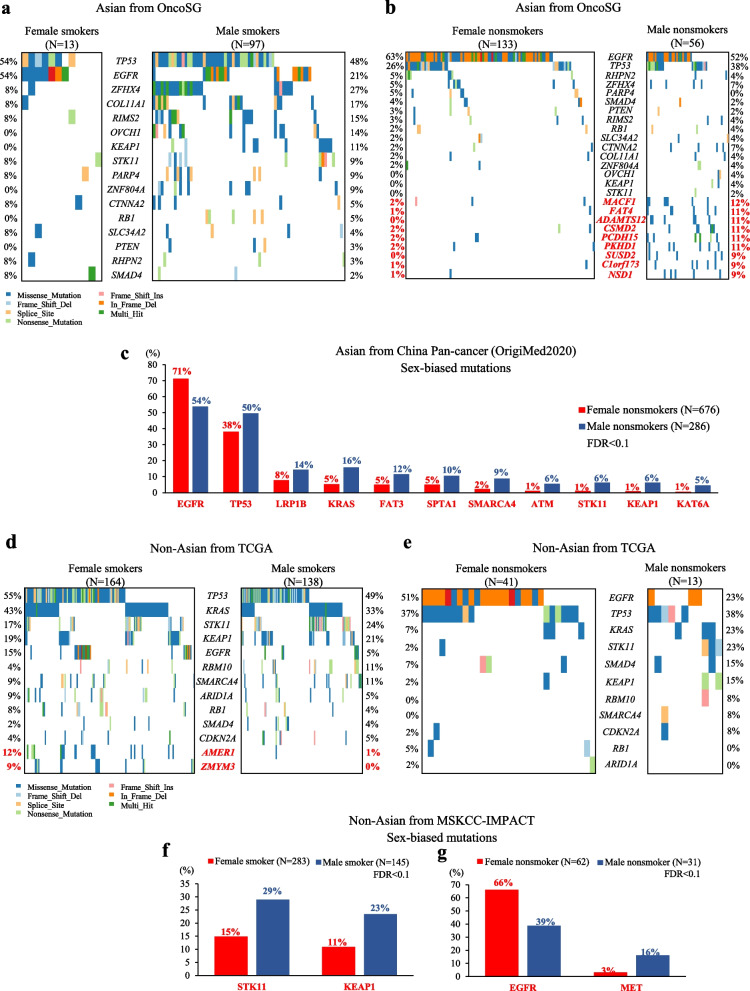
Sex-biased genetic alterations in LUADs. **a** Mutation landscape of driver mutations between Asian female and male smokers with LUAD in the OncoSG cohort. **b** Mutation landscape of driver mutations (black font) and sex-biased mutations (red font) between Asian female and male nonsmokers with LUAD. **c** Sex-biased mutations between Asian female and male nonsmokers with LUAD in the OrigiMed2020 cohort. **d** Driver mutations (black font) and sex-biased mutations (red font) between sexes in Non-Asian LUAD smokers from TCGA cohort. **e** Driver mutations between sexes in Non-Asian LUAD nonsmokers from TCGA cohort. **f** Sex-biased mutations between Non-Asian female and male LUAD smokers in the MSKCC-IMPACT cohort. **g** Sex-biased mutations between Non-Asian female and male LUAD nonsmokers in the MSKCC-IMPACT cohort

In Asian LUAD patients (OncoSG), we identified 16 driver mutations using MutSigCV algorithm (FDR < 0.05) (Additional file 1: Table S4). Next, we focused on the genes with driver mutations as well as genes with a mutation frequency higher than 2% across Asian LUAD patients, and compared the mutation patterns between male and female patients. To control the impact of smoking on the genetic variants, we performed a stratification analysis by smoking status. At FDR < 0.1, no sex differences were observed in driver mutations in Asian LUAD patients irrespective of smoking status (Fig. 1a-b, Additional file 1: Table S5-S6). However, when analysis was extended to non-driver mutations, nine male-biased mutations were identified in Asian nonsmokers (Fig. 1b, Additional file 1: Table S6). Similarly, the silico analysis of deep targeted NGS data covering 450 cancer-related genes (OrigiMed2020 cohort) defined 11 sex-biased mutations in Asian LUAD nonsmokers (Fig. 1c, Additional file 1: Table S7). All of these but *EGFR* were identified as male-biased mutations, including *TP53*, *LRP1B*, *KRAS*, *FAR3*, *SPTA1*, *SMARCA4*, *ATM*, *STK11*, *KEAP1*, and *KAT6A* (Fig. 1c), but these sex divergences were not observed in Asian smokers (data not shown). Altogether, male-biased mutations were prevalent in Asian nonsmoking LUADs, while no significant sex differences in mutations were observed in Asian smoking LUADs.

In Non-Asian LUAD patients (TCGA cohort), we identified 11 driver mutations (Additional file 1: Table S8), while no sex differences were observed in these driver mutations regardless of cigarette smoking (Fig. 1d-e, Additional file 1: Table S9-S10). Of note, non-driver mutations such as *AMER1* (12% vs. 1%, FDR = 0.081) and *ZMYM3* (9% vs. 0%, FDR = 0.081) mutations showed a significant higher frequency in female smokers compared to male smokers (Fig. 1d), whereas none of genetic mutations showed sex biases in nonsmokers (Fig. 1e). Additionally, in MSKCC-IMPACT cohort, *STK11* (29% vs. 15%, FDR = 0.077) and *KEAP1* (23% vs. 11%, FDR = 0.077) mutations occurred more frequently in male smokers compared with female smokers (Fig. 1f, Additional file 1: Table S11). Among nonsmoking LUADs, the incidence of *EGFR* mutation was significantly higher in females than in males (66% vs. 39%, FDR = 0.088), while higher mutation frequency of *MET* was observed in males than in females (16% vs. 3%, FDR = 0.088) (Fig. 1g, Additional file 1: Table S12).

These results suggested that genetic alterations in cancer are sexually dimorphic, and in the different contexts of ethnicity, the smoke-related genotoxicity might lead to largely contrasting consequence, with or without respect to sex.

### Sex-biased expression profiles of genes from autosomal and sex chromosomes

**Fig. 2:**
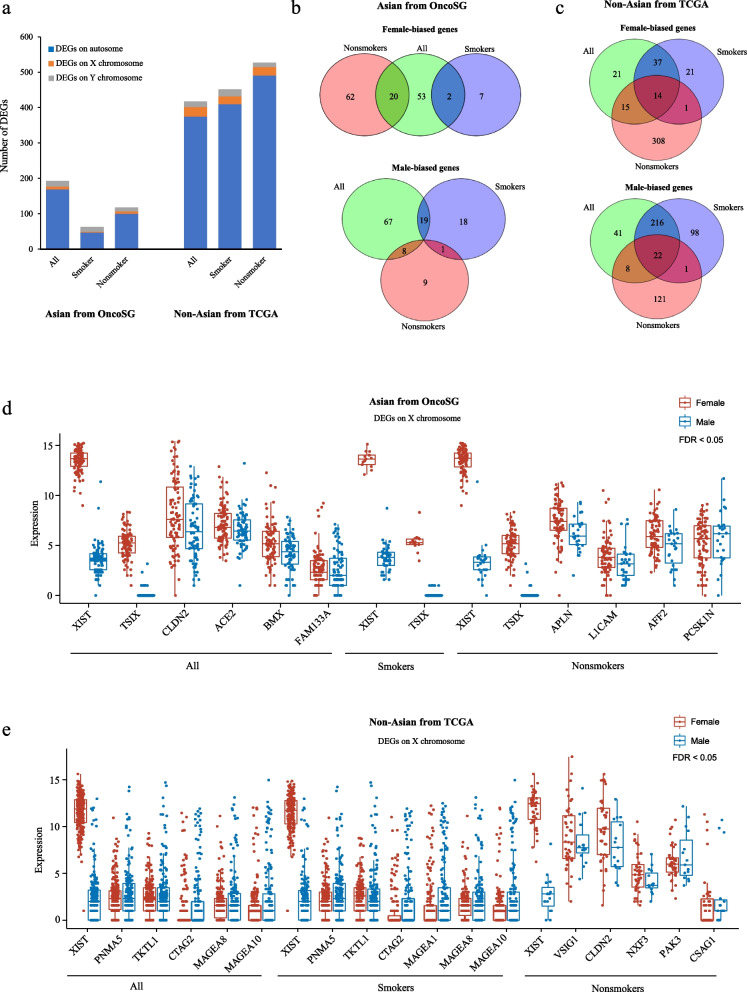
Sex-biased gene expression in LUADs. **a** The number of DEGs in Asian and Non-Asian LUADs. **b**-**c** The number of sex-biased genes on autosomal chromosomes. **d**-**e** Expression levels of sex-biased genes on X chromosomes. All genes were DEGs (|log2 fold change|> 1 and FDR < 0.05)

When compared to Asian LUADs, Non-Asian LUADs exhibited a larger number of DEGs between two genders (Fig. 2a). Next, we focused on sex-specific genes on autosomal chromosomes. In Asian cohort, none or few overlapping sex-biased genes were observed between smokers and nonsmokers (Fig. 2b, Additional file 2: Table S13, Additional file 3: Table S14, and Additional file 4: Table S15). In Non-Asian cohort, the number of female-biased genes on autosome chromosome was greater than that of male-biased genes in LUAD nonsmokers (339 vs. 152 genes), whereas the opposite results were observed in LUAD smokers (73 vs. 337 genes) (Fig. 2c, Additional file 5: Table S16, Additional file 6: Table S17, and Additional file 7: Table S18). Additionally, in Asian population, regardless of smoking status, genes originated from the X chromosomes showed female-biased expression (Fig. 2d). For instance, *XIST* expression was significantly higher in females than males, consistent with previous reports [28]. However, some X chromosome-located genes showed higher expression in Non-Asian males compared to females (Fig. 2e), but this pattern was not detected in Asian LUADs. We found that the expression of type I melanoma associated antigens (MAGEs), including *MAGEA1*, *MAGEA8*, and *MAGEA10*, was increased in male smokers with LUAD (Fig. 2e). MAGE family members, specifically type I MAGE, are regarded as cancer testis antigens, and they serve important roles in tumorigenesis and cancer cell survival [29].

### Sex-biased activity of biological pathways

**Fig. 3:**
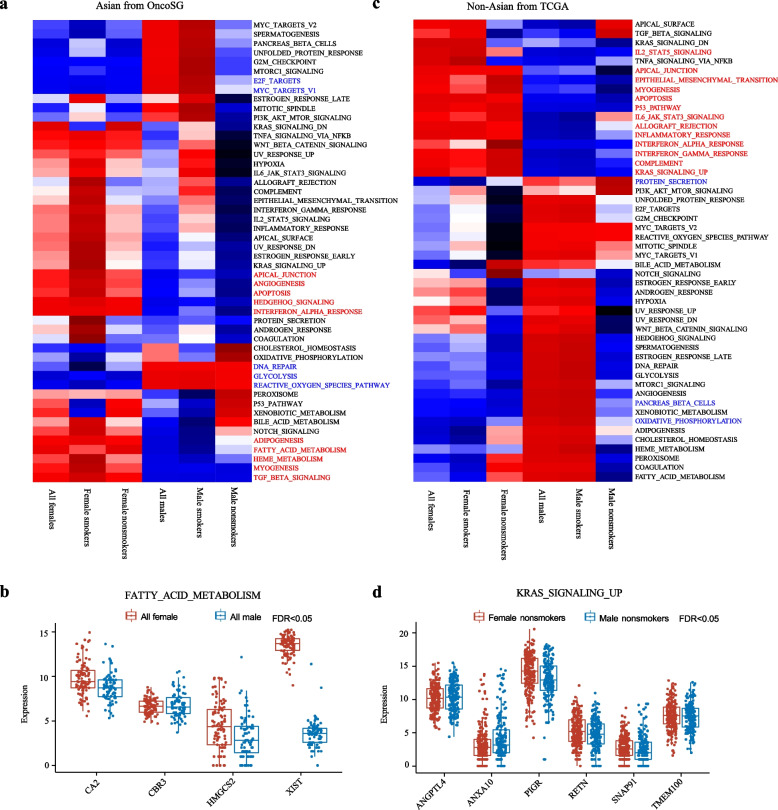
Sex-biased biological pathway in LUADs. **a** The heatmaps showing the sex-biased pathways in the Asian population. **b** Expression levels of sex-biased genes involve in the FATTY_ACID_METABOLISM pathway. **c** The heatmaps showing sex-biased pathways in the Non-Asian population. **d** Expression levels of sex-biased genes involved in KRAS_SIGNALING_UP pathway. Red font: female-biased pathways. Black font: male-biased pathways

Next, we performed ssGSEA based on total gene expression to assess biological pathways distinctly enriched in different sexes. As expected, female and male LUADs showed distinct patterns of hallmark pathways (Fig. 3). In Asian cohort, gene sets related to angiogenesis, apoptosis, interferon_alpha_response, and fatty_acid_metabolism, showed higher activity in female LUADs, whereas gene sets related to E2F_targets, MYC_targets_v1, and DNA_repair were highly expressed in male LUADs (Fig. 3a). Of note, E2F_ targets and MYC_ targets _v1 exhibited enrichment only in male smokers, but not in nonsmokers. Similarly, the comparison at individual gene expression levels showed that genes involved in fatty_acid_metabolism such as *CA2*, *CBR3*, *HMGCS2*, and *XIST* were significantly upregulated in Asian females (Fig. 3b). In Non-Asian cohort, immune-related pathways were significantly enriched in females, including inflammatory_response, interferon_alpha_response, interferon_gamma_response, and IL6-JAK-STAT3 signaling (Fig. 3c). We also found significant enrichment of KRAS signaling (KRAS_SIGNALING_UP) in females (Fig. 3c-d). These results might suggest that in cancer cells biological processes were differentially activated between female and male LUADs.

### Sex-biased tumor-infiltrating immune cell

**Fig. 4:**
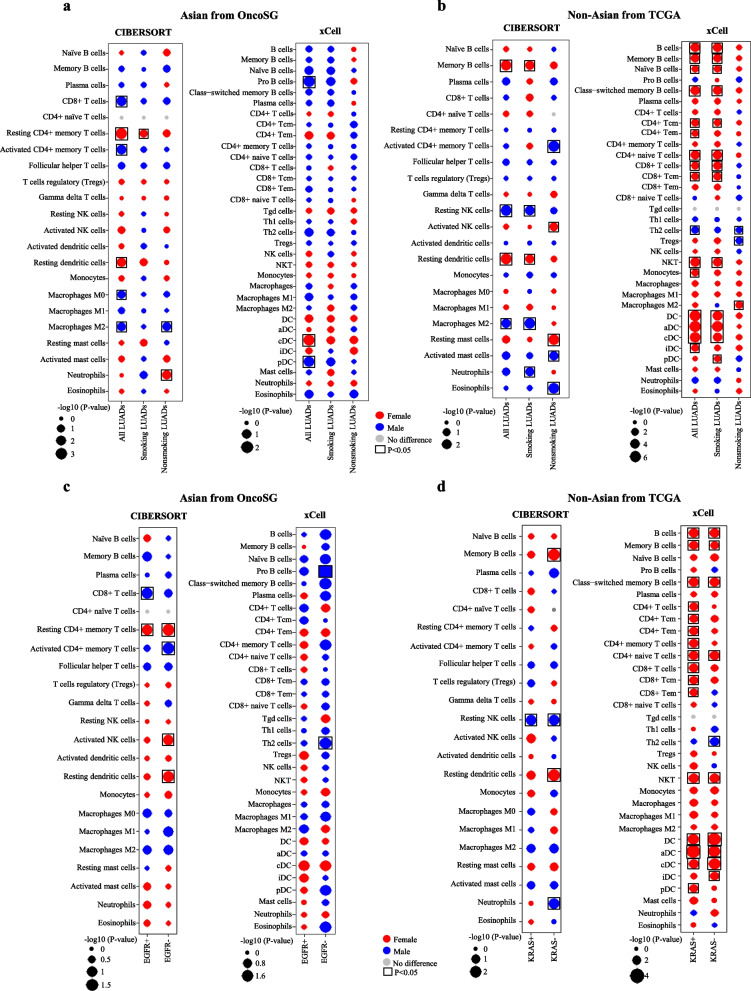
Sex-biased immune cells in LUADs. **a**-**b** The bubble plots display the distribution of immune cells between female and male LUADs in (**a**) the Asian and (**b**) the Non-Asian cohorts using CIBERSORT and xCell methods. **c**-**d** The bubble plots display the distribution of immune cells between sexes in (**c**) *EGFR* mutant and wild-type Asian LUADs and in (**d**) *KRAS* mutant and wild-type Non-Asian LUADs. The black boxes represent a statistical significance from Mann–Whitney U test (*P* < 0.05). DC: Dendritic cells, aDC: Activated dendritic cells, cDC: Conventional dendritic cells, iDC: Immature dendritic cells, pDC: Plasmacytoid dendritic cells

To study sex disparities in immune cell populations in tumor microenvironment, we used two approaches to assess and compare the immune infiltration between female and male patients with respect to ethnicity and smoking status. In the Asian LUADs (OncoSG cohort), we found by using CIBERSORTx approach that tumors from all female LUADs presented significantly more resting CD4 + memory T cells and resting dendritic cells irrespective of smoking status, whereas tumors from all male LUADs were more enriched with CD8 + T cells, activated CD4 + memory T cells, and M0/M2 macrophages (Fig. 4a). And the pattern of those immune cell infiltration observed in the OncoSG cohort was further confirmed in the CHOICE cohort (Additional file 1: Figure S1). The tendency of most sex-biased immune infiltration detected by xCell was same as  that detected by CIBERSORTx, although the trend was not statistically significant in both employed methods. Nevertheless, above-described immune infiltration patterns were recapitulated in both stratified smokers and nonsmokers (Additional file 1: Figure S1). However, we observed also inconsistent results between two approaches. Reported by CIBERSORTx, female patients had more infiltration of resting CD4 + memory T cells, while male patients had more abundant activated CD4 + memory T cells (Additional file 1: Figure S1). But xCell reported more abundant CD4 + memory T cells in male LUADs (Additional file 1: Figure S1). We speculated that this disparity resulted from the fact that different subsets of CD4 + memory T cells were analyzed by two programs. xCell enumerated only CD4 + memory T cells but CIBERSORT dissected the latter further into functional subsets: resting and active CD4 + memory T cells. Further independent validation analyses and experiments should be performed to verify the cellular heterogeneity of CD4 + memory T cells in LUADs.

In the Non-Asian population, we found smoking status exerted great impact on sex-biased immune infiltration (Fig. 4b). Of note, tumors in female smokers were more infiltrated with memory B cells (or B cells), resting dendritic cells, and CD8 + T cells, whereas male smokers had more infiltration of M2 macrophages and neutrophils. In contrast, among nonsmokers, M2 macrophages were more abundant in females than males. LUADs derived from males presented more abundant resting NK cells and Th2 cells, regardless of smoking status.

It has been reported that genetic alterations were associated with differential composition of immune cells in tumor microenvironment [30, 31]. In the current study, CD8 + T cells were more abundant in male patients with *EGFR* mutation-positive LUAD compared to females, whereas resting CD4 + memory T cells followed the opposite pattern, although it was only statistically significant in the results obtained from CIBERSORT analysis (Fig. 4c). More importantly, LUADs harboring wild-type *EGFR* in female patients had a significantly increased fraction of resting CD4 + memory T cells, activated NK cells, and resting dendritic cells, as well as a significantly declined fraction of activated CD4 + memory T cells, compared to the same type of LUADs in male patients (Fig. 4c). We also observed a higher level of B cells infiltration in females compared to males regardless of *KRAS* genotype, whereas an opposite trend was observed in which resting NK cells were more abundant in male LUADs (Fig. 4d). Additionally, we also detected that CD8 + and CD4 + T cells were more abundant in female LUADs harboring mutant *KRAS* compared to male counterparts (Fig. 4d).

### Exclusive correlation between SCGB3A2 high expression and response to ICB in female LUADs

**Fig. 5:**
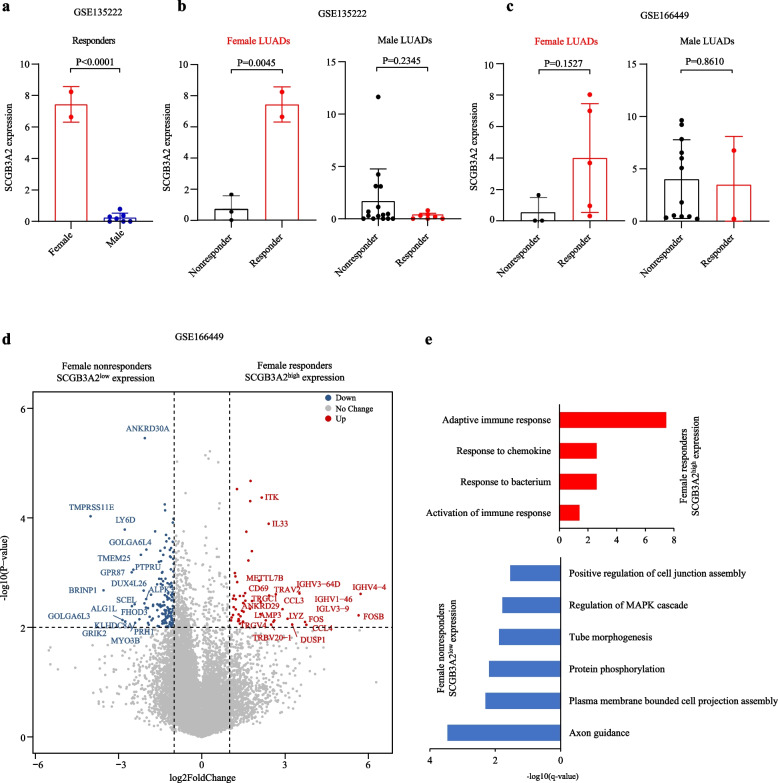
The correlation between SCGB3A2 expression and response to ICB in female LUADs. **a** The expression levels of SCGB3A2 between female and male responders to ICB. **b**-**c** The expression levels of SCGB3A2 between responders and nonresponders. **d** Volcano plot showed the DEGs between female responders with SCGB3A2^high^ expression and female nonresponders with SCGB3A2^low^ expression. **e** Significantly enriched pathways in the DEGs

It is clinically established that female patients respond to cancer immunotherapy better than male patients [4, 32–34]. To explore molecular determinants for the differences in response to immunotherapy between female and male LUADs, the transcriptomic profiles of LUAD tumors who were sensitive to ICB treatment were obtained from GSE135222 and GSE166449 datasets. The detailed analyses revealed that the gene expression profiles of female and male responders to ICB differed in very few genes in GSE135222 dataset (Additional file 8: Table S19). Among these genes, secretoglobin family 3A member 2 (*SCGB3A2*) is the most significantly upregulated DEGs in female responders compared with male responders (Fig. 5a). Interestingly, we observed that all female patients with high *SCGB3A2* expression were exclusively sensitive to ICBs, while this phenomenon was not observed in male patients (Fig. 5b). And this exclusive association was validated in an independent cohort (GSE166449) in which all *SCGB3A2*-high female LUADs were ICB responders (Fig. 5c). Further analysis of the expression profiles of *SCGB3A2*-high female responders disclosed that these ICB sensitive tumors exhibited distinguishing high expression of genes typifying the activation of T and B cells, including *CD69*, *CCL4*, *ITK*, *IL33*, IGHV family members (Fig. 5d) and the high activity of pathways related to adaptive immune response (Fig. 5e).

## Discussion

As evidenced by pioneer studies and our preliminary analyses mentioned hereabove, the molecular factors, such as genetic and molecular alterations, extensively engage in, quantitively and functionally, shaping sex-based disparity in cancer etiology, progression, personalized therapy, and prognosis. Unlike other determinants for sex-disparities such as sociodemographic and lifestyle factors, intrinsic genetic and epigenetic scenario have been rarely investigated but definitely are underestimated. In the current study, we found that distinct ethnic background, smoking status, and genetic alterations largely complexified the disparities in genomic profiles, gene expressions and immunological landscapes between sexes in LUAD. This study focused on lung adenocarcinoma, but doubtlessly it is highly possible that our findings and hypotheses can be recapitulated and tested in other subtypes of lung cancer as well as other cancer types.

**Fig. 6:**
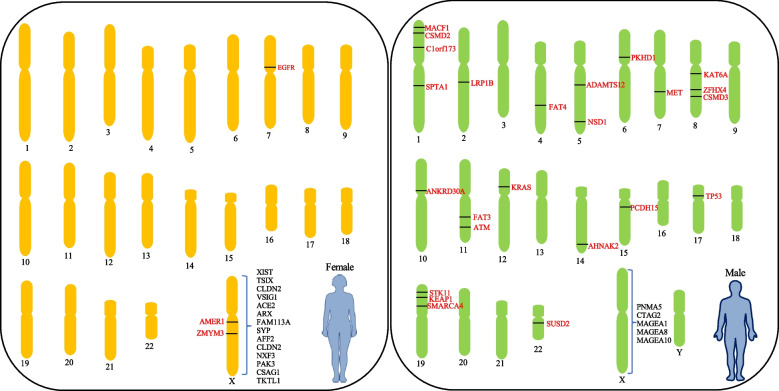
Outline of main differences in genetic alteration and gene expression in X chromosomes between the sexes from all LUAD patients in this study. These genes are simply mapped on chromosomes. Red font: mutated genes. Black font: upregulated genes in X chromosomes

The comparative genetic analysis of female and male LUADs revealed a different distribution of mutations between female and male LUADs. As illustrated in Fig. 6, female-biased mutations except for *EGFR* were predominantly detected on X chromosome whereas male-biased mutations found on autosomal chromosomes. Generally, in LUADs male-dominant genetic mutations found in both oncogenes, such as *KRAS* and *MET*, as well as genes associated with broad biological functions, such as *STK11*, *KEAP1*, and *LRP1B* genes. In a further refined analysis bringing into the potential impact of smoking status, a higher frequency of *EGFR* mutation was observed in nonsmoking females compared with male nonsmokers, consistent with previous study [35, 36].

One interesting observation from the current study is, genetic alterations rarely show statistically significant differences between sexes in smoking Asian LUADs, while substantial genetic alterations display sex differences in non-smoking Asian LUADs. In non-Asian patients, sex-biased mutations were prevalent in both smoking and non-smoking LUADs. These phenomena indicate that smoke-related carcinogens exert very broad effect on genes with fundamental functions in both female and male patients, which overwhelms or masks the sex-biased genetic alterations induced by other cancer risk factors. Hence, we advocate that LUAD should be studied as a different disease specific to ethnicity and etiology such as different smoking history, especially when implementing screening or diagnosis programs and clinical trial.

At transcriptome level, we found female LUADs were characterized by activated immune pathways, whereas male LUADs by DNA repair and E2F_targets signaling pathways. Although the difference of gene expression by sex in LUADs has not been well documented, differential gene expressions and activity of biological pathways have been previously reported in other cancer types [8]. Expression analysis in bladder cancer confirmed that increased expression of basal- and immune-associated genes was observed in female tumors, while male tumors expressed higher levels of luminal markers [37]. In hepatocellular carcinoma, female subjects were enriched with PPAR pathway, whereas males with PI3K, PI3K/AKT, FGFR, EGFR, and IL-2 signaling pathway [38]. Moreover, the scale of sex-biased aberrant expression further varies by race in LUADs. For example, Non-Asian LUADs had a larger number of sex-biased genes compared with Asian LUADs.

Sex differences in immune microenvironment have been found in multiple types of cancer. For example, female patients with lung squamous cell carcinoma had significantly higher abundance of activated CD4 + T cells and activated CD8 + T cells [32]. While in kidney renal papillary cell carcinoma, a study described a general higher density of immune cells and mRNA expression of immune checkpoints in tumor microenvironment in male than in female cases [32]. Our analysis also deconvolved that the infiltration of resting CD4 + memory T cells, B cells, and resting dendritic cells in female LUADs was higher than those in male LUADs. Contrarily, CD8 + T cells, activated CD4 + memory T cells, and M0/M2 macrophages were more abundant in male LUADs in comparison to female LUADs. These findings might provide important theoretical basis for explaining the relatively superior prognosis and therapeutic response of female LUADs, but more investigation and mechanistic experiments are necessary.

Different trends of genetic alterations by sex have been reported and linked to sex-specific immune infiltration. For instance, the loss of *PTEN* was found in prostate cancers with more infiltration of Gr-1 + CD11b + myeloid cells (immature myeloid cells, monocytes, and neutrophils) [30]. In a similar study, loss of *PTEN* was speculated to be one of the reasons of reduced CD8 + T cell infiltration in melanoma models [31]. In the current study, male LUADs harboring mutant *EGFR* had a significantly increased infiltration of CD8 + T cells, whereas resting CD4 + memory T cells were more abundant in female LUADs with *EGFR* mutation. These observations highly indicated the existence of cause-and-effect relationship between oncogenic alterations and immune infiltration, and these events collectively might make cancer cells more susceptible to immunotherapy. However, there are still no conclusive data to verify these speculations due to the lack of study materials for simultaneous genetic and immunological profiling from LUADs patients who are treated with anti-PD-1 or anti-PD-L1-based monotherapy.

In this study, we detected an exclusive association between high expression of *SCGB3A2* and response to ICB treatment in female LUADs, although the number of responders is small. However, the association was retained in an independent LUAD cohort treated with ICB. It would be important to further verify this exclusive association demonstrated by these female responders when more genomic data of ICB-treated LUAD patients are available in future. *SCGB3A2* is predominantly expressed in the lung airway epithelial cells [39, 40]. Previous study has demonstrated that LUAD cells with highly expressed *SCGB3A2* exhibited anti-inflammatory features [41], it was not clarified whether this was related to the enhanced efficacy of immunotherapy. Furthermore, *SCGB3A2* is identified as a downstream target for the homeodomain transcription factor NK2 Homeobox 1 (*NKX2-1*) (also known as *TTF1*) [42], which is utilized as a marker for LUAD diagnosis [43]. Notably, recent studies revealed that patients with TTF-1-positive status receiving immune-checkpoint inhibitor monotherapy showed better outcome than those with TTF-1-negative LUAD [44]. However, whether the interplay between *SCGB3A2* and *TTF-1* could further enhance the efficacy of immunotherapy requires further research. The successful validation of these observations might allow more precise selection schemes to find female LUADs who will benefit from ICB treatment.

Not limited to genetic variants, gene expression, and immune features, the epigenetics, or chromatin conformation might also have fundamental, even more important roles in shaping sexual dimorphisms in cancer biology, which should not be underestimated, although we did not further investigate these in the current study. Sex differences in the epigenomic landscape have been reported in many cancers. A recent study identified 1043 sex-biased CpG sites in chronic lymphocytic leukemia [45]. Recent studies on sex-specific chromatin accessibility have shown that B cell-specific loci were more likely in an opening status in women and a closing status in men [46]. Sex differences in epigenetics could predispose the expression of related genes to a sex-biased mode, as evidence by the observation of the differentially expressed genes between men and women resulting from sex-specific DNA methylation and chromatin accessibility [8, 47, 48]. Furthermore, understanding the collective effect of these intrinsic and external determinants could better clarify the phenotypic sex differences in cancers [49]. A recent study on the prognosis of lung cancer has shown that female LUADs characterized by wild-type *TP53*, high levels of immune infiltration, and enrichment of immune-related pathways or compartments such as INF-γ and TNF signaling and macrophages-monocytes obtained longer survival, whereas male with lung squamous cell carcinoma, wild-type *TP53* tumor cells, and enriched TGF-β signaling pathway showed poor prognosis [50]. To obtain a complete picture of sex-related cancer biology, future studies should be thoroughly conducted at multi-omics levels and integrate multi-omics data, which is able to pave a solid foundation for the development of sex-stratified therapeutic and patient managemental strategies. Importantly, the role of sex should also be considered when preclinical and clinical research is performed. Related considerations include the sex of an experimental model, the enrollment into clinical trials, and distinct biomarkers used for men and women.

Our study has a few limitations. First, the analyzed data was retrieved from the public databases. Due to insufficient clinical information, we were unable to investigate the effect of some important physiopathological factors on sex differences in LUAD, including hormone status, endocrine history, body mass index, and therapeutic history, warranting the need for clinical studies in larger patient cohorts with much more complete clinical information in future. Second, computational deconvolution algorithms, including the ones used in our study, deduce the immunophenotypes based on bulk cell sequencing data and only can suggest the relative abundance of pre-selected types of immune cells. Therefore, further experiments such as multiplex immunohistochemistry/immunofluorescence and single-cell RNA-sequencing need to be performed to confirm the inferred differences in tumor immune microenvironment between sexes.

## Supplementary Information

**Additional file 1:** **Table S1.** Summary of data sets used in this study. **Table S2.** The clinical characteristics of Asian LUAD patients for analyzing genetic variants in this study. **Table S3.** The clinical characteristics of Non-Asian LUAD patients for analyzing genetic variants in this study. **Table S4.** The driver mutations in the OncoSG cohort. **Table S5.** Comparison of driver mutations between female and male smokers with LUAD in the OncoSG cohort. **Table S6.** Comparison of driver and passenger mutations between female and male nonsmokers with LUAD in the OncoSG cohort. **Table S7.** Comparison of driver mutations between female and male smokers with LUAD in the OrigiMed2020 cohort. **Table S8.** The driver mutations in the TCGA cohort. **Table S9.** Comparison of driver and passenger mutations between female and male smokers with LUAD in the TCGA cohort. **Table S10.** Comparison of driver mutations between female and male nonsmokers with LUAD in the TCGA cohort. **Table S11.** Comparison of driver mutations between female and male smokers with LUAD in the MSKCC-IMPACT cohort. **Table S12.** Comparison of driver mutations between female and male nonsmokers with LUAD in the MSKCC-IMPACT cohort.** Figure S1.** The distributions of immune cell subtypes between male and female LUADs.

**Additional file 2:** **Table S13.** The DEGs identified from the comparison between all female and male LUADs in the OncoSG cohort (Asian).

**Additional file 3:** **Table S14.** The DEGs identified from the comparison between female and male non-smokers with LUAD in the OncoSG cohort  (Asian).

**Additional file 4:** **Table S15.** The DEGs identified from the comparison between female and male smokers with LUAD in the OncoSG cohort (Asian).

**Additional file 5:** **Table S16.** The DEGs identified from the comparison between all female and male LUADs in the TCGA cohort (Non-Asian).

**Additional file 6:** **Table S17.** The DEGs identified from the comparison between female and male non-smokers with LUAD in the TCGA cohort (Non-Asian).

**Additional file 7:** **Table S18.** The DEGs identified from the comparison between female and male smokers with LUAD in the TCGA cohort (Non-Asian).

**Additional file 8:** **Table S19.** The DEGs identified from the comparison between female and male responders to ICB in the GSE135222 dataset.

## Data Availability

The data that support the findings of this study are available from public data repositories, including the OncoSG (https://src.gisapps.org/OncoSG/), OrigiMed2020 (http://www.cbioportal.org), CHOICE (https://doi.org/10.6084/m9.figshare.7306364.v1), TCGA (https://portal.gdc.cancer.gov/), MSK-IMPACT (http://www.cbioportal.org), GSE135222 (https://www.ncbi.nlm.nih.gov/geo/query/acc.cgi?acc=GSE135222), and GSE166449 (https://www.ncbi.nlm.nih.gov/geo/query/acc.cgi?acc=GSE166449) cohorts (Additional file 1: Table S1).

## Abbreviations

LUAD: Lung adenocarcinoma
NSCLC: Non-small cell lung cancer
NGS: Next-generation sequencing
TCGA: The Cancer Genome Atlas
FDR: False discovery rate
DEGs: Differentially expressed genes
ssGSEA: Single-sample gene set enrichment analysis
MSigDB: Molecular Signatures Database
MAGEs: Melanoma associated antigens
SCGB3A2: Secretoglobin family 3A member 2
ICB: Immune chokepoint inhibitor
NKX2-1: NK2 Homeobox 1
TTF-1: Thyroid transcription factor 1
