# Supplementary material for: Sex-biased molecular differences in lung adenocarcinoma are ethnic and smoking specific
Source: BMC Pulm Med. 2023 Mar 24;23:99. doi: 10.1186/s12890-023-02387-7 (PMC10039609; doi:10.1186/s12890-023-02387-7)
Supplement: Supplementary file 1 — Additional file 1: Table S1. Summary of data sets used in this study. Table S2. The clinical characteristics of Asian LUAD patients for analyzing genetic variants in this study. Table S3. The clinical characteristics of Non-Asian LUAD patients for analyzing genetic variants in this study. Table S4. The driver mutations in the OncoSG cohort. Table S5. Comparison of driver mutations between female and male smokers with LUAD in the OncoSG cohort. Table S6. Comparison of driver and passenger mutations between female and male nonsmokers with LUAD in the OncoSG cohort. Table S7. Comparison of driver mutations between female and male smokers with LUAD in the OrigiMed2020 cohort. Table S8. The driver mutations in the TCGA cohort. Table S9. Comparison of driver and passenger mutations between female and male smokers with LUAD in the TCGA cohort. Table S10. Comparison of driver mutations between female and male nonsmokers with LUAD in the TCGA cohort. Table S11. Comparison of driver mutations between female and male smokers with LUAD in the MSKCC-IMPACT cohort. Table S12. Comparison of driver mutations between female and male nonsmokers with LUAD in the MSKCC-IMPACT cohort. Figure S1. The distributions of immune cell subtypes between male and female LUADs. [file 12890_2023_2387_MOESM1_ESM.pdf]

Supplemental Tables and Figures

Table S1. Summary of data sets used in this study.

Cohort	Race	Somatic mutation	Method of DNA sequencing	mRNA expression	Method of RNA sequencing
**OncoSG**	Asian	299	WES	167	RNA-seq
**OrigiMed2020**	Asian	1370	Targeted NGS	-	-
**CHOICE**	Asian	-	-	128	RNA-seq
**TCGA**	Non-Asian	356	WES	356	RNA-seq
**MSK-IMPACT**	Non-Asian	521	Targeted NGS	-	-
**GSE135222**	Asian	-	-	27	RNA-seq
**GSE166449**	Asian	-	-	22	RNA-seq

Table S2. The clinical characteristics of Asian LUAD patients for analyzing genetic variants in this study.

Characteristics	OncoSG (Asian)						OrigiMed2020 (Asian)					
**Sex**	Female	Male	*P*	Female	Male	*P*	Female	Male	*P*	Female	Male	*P*
**Smoking status**	smoker	smoker		nonsmoker	nonsmoker		smoker	smoker		nonsmoker	nonsmoker	
**Total**	13	97		133	56		18	390		676	286	
**Age**												
**<65**	6	45	*1*	77	28	*0.403*	10	242	*0.759*	493	204	*0.668*
**≥65**	7	52		56	28		8	148		183	82	
**Stage**												
**I**	8	41	*0.492*	69	18	*0.077*	6	127	*0.360*	319	122	*0.054*
**II**	2	18		21	15		4	39		72	20	
**III**	3	29		36	20		2	82		95	55	
**IV**	0	9		7	3		6	142		190	88	

P: Chi-squared test

Table S3. The clinical characteristics of Non-Asian LUAD patients for analyzing genetic variants in this study.

Characteristics	TCGA (Non-Asian)						MSK-IMPACT (Non-Asian)					
**Sex**	Female	Male	*P*	Female	Male	*P*	Female	Male	*P*	Female	Male	*P*
**Smoking status**	smoker	smoker		nonsmoker	nonsmoker		smoker	smoker		nonsmoker	nonsmoker	
**Total**	164	138		41	13		283	145		62	31	
**Age**												
**<65**	86	60	*0.151*	14	4	*1*	97	28	***0.002***	25	11	*0.821*
**≥65**	78	78		27	9		186	117		37	20	
**Stage**												
**I**	101	71	*0.072*	21	6	*0.75*	-	-	-	-	-	-
**II**	31	38		13	6		-	-		-	-	
**III**	28	20		6	1		-	-		-	-	
**IV**	4	9		1	0		-	-		-	-	

P: Chi-squared test

Table S4. The driver mutations in the OncoSG cohort.

Gene	N_nonsilent	N_silent	P value	FDR
EGFR	155	6	0	0
SMAD4	11	0	0	0
TP53	112	1	0	0
RHPN2	14	0	2.71E-09	1.02E-05
RB1	10	1	2.27E-08	7.15E-05
PARP4	16	1	4.36E-07	0.001
PTEN	8	0	8.38E-07	0.002
OVCH1	17	0	1.20E-06	0.003
RIMS2	26	3	1.74E-06	0.003
SLC34A2	9	0	5.27E-06	0.009
CTNNA2	12	2	8.79E-06	0.014
KEAP1	13	1	1.55E-05	0.022
ZFHX4	47	10	1.73E-05	0.023
COL11A1	24	3	2.00E-05	0.025
STK11	11	2	2.24E-05	0.026
ZNF804A	14	2	3.07E-05	0.034

Table S5. Comparison of driver mutations between female and male smokers with LUAD in the OncoSG cohort.

Gene	Female	Male	P value	FDR	Driver mutation	Sex-biased
TP53	7 (54%)	46 (48%)	0.773772	1	Yes	Unbiased
EGFR	7 (54%)	20 (21%)	0.017173	1	Yes	Unbiased
ZFHX4	1 (8%)	26 (27%)	0.178113	1	Yes	Unbiased
COL11A1	1 (8%)	16 (17%)	0.687454	1	Yes	Unbiased
RIMS2	1 (8%)	14 (15%)	0.68916	1	Yes	Unbiased
OVCH1	0 (0%)	13 (14%)	0.359192	1	Yes	Unbiased
KEAP1	0 (0%)	11 (11%)	0.353556	1	Yes	Unbiased
STK11	1 (8%)	9 (9%)	1	1	Yes	Unbiased
PARP4	1 (8%)	9 (9%)	1	1	Yes	Unbiased
ZNF804A	0 (0%)	9 (9%)	0.596011	1	Yes	Unbiased
CTNNA2	1 (8%)	5 (5%)	0.545815	1	Yes	Unbiased
RB1	0 (0%)	5 (5%)	1	1	Yes	Unbiased
SLC34A2	1 (8%)	4 (4%)	0.476804	1	Yes	Unbiased
PTEN	0 (0%)	3 (3%)	1	1	Yes	Unbiased
RHPN2	1 (8%)	3 (3%)	0.402874	1	Yes	Unbiased
SMAD4	1 (8%)	2 (2%)	0.319405	1	Yes	Unbiased

Table S6. Comparison of driver and passenger mutations between female and male nonsmokers with LUAD in the OncoSG cohort.

Gene	Female	Male	P value	FDR	Driver mutation	Sex-biased
EGFR	81 (63%)	29 (52%)	0.190929	0.375275	Yes	Unbiased
TP53	33 (26%)	21 (38%)	0.116551	0.331138	Yes	Unbiased
RHPN2	7 (5%)	2 (4%)	0.724707	0.841144	Yes	Unbiased
ZFHX4	7 (5%)	4 (7%)	0.737846	0.841144	Yes	Unbiased
PARP4	6 (5%)	0 (0%)	0.179911	0.366246	Yes	Unbiased
SMAD4	5 (4%)	1 (2%)	0.669028	0.841144	Yes	Unbiased
PTEN	4 (3%)	1 (2%)	1	1	Yes	Unbiased
RIMS2	4 (3%)	2 (4%)	1	1	Yes	Unbiased
RB1	3 (2%)	2 (4%)	0.636606	1	Yes	Unbiased
SLC34A2	2 (2%)	2 (4%)	0.584369	1	Yes	Unbiased
CTNNA2	2 (2%)	4 (7%)	0.066832	0.8212	Yes	Unbiased
COL11A1	2 (2%)	2 (4%)	0.584369	1	Yes	Unbiased
ZNF804A	2 (2%)	2 (4%)	0.584369	1	Yes	Unbiased
KEAP1	0 (0%)	2 (4%)	0.088552	0.8212	Yes	Unbiased
OVCH1	0 (0%)	2 (4%)	0.088552	0.8212	Yes	Unbiased
STK11	0 (0%)	1 (2%)	0.299465	0.8212	Yes	Unbiased
MACF1	3 (2%)	7 (12%)	0.009597	0.066904	No	Male-biased
FAT4	1 (1%)	6 (11%)	0.003476	0.06605	No	Male-biased
ADAMTS12	0 (0%)	6 (11%)	0.000654	0.037284	No	Male-biased
CSMD2	2 (2%)	6 (11%)	0.010564	0.066904	No	Male-biased
PCDH15	2 (2%)	6 (11%)	0.010564	0.066904	No	Male-biased
PKHD1	2 (2%)	6 (11%)	0.010564	0.066904	No	Male-biased
SUSD2	0 (0%)	5 (9%)	0.002296	0.06543	No	Male-biased
C1orf173	1 (1%)	5 (9%)	0.010504	0.066904	No	Male-biased
NSD1	1 (1%)	5 (9%)	0.010504	0.066904	No	Male-biased

Table S7. Comparison of driver mutations between female and male smokers with LUAD in the OrigiMed2020 cohort.

Gene	Female	Male	P value	FDR	Driver mutation	Sex-biased
EGFR	482 (71%)	154 (54%)	1.73E-07	7.54E-05	Yes	Female-biased
KRAS	36 (5%)	45 (16%)	3.51E-07	7.65E-05	Yes	Male-biased
KEAP1	6 (1%)	18 (6%)	4.60E-06	6.68E-04	Yes	Male-biased
STK11	7 (1%)	18 (6%)	1.20E-05	1.31E-03	Yes	Male-biased
SMARCA4	16 (2%)	25 (9%)	2.17E-05	1.89E-03	Yes	Male-biased
KAT6A	4 (1%)	13 (5%)	8.02E-05	5.83E-03	Yes	Male-biased
ATM	8 (1%)	16 (6%)	1.67E-04	0.0104	Yes	Male-biased
FAT3	34 (5%)	33 (12%)	3.87E-04	0.0211	Yes	Male-biased
TP53	258 (38%)	142 (50%)	6.38E-04	0.029	Yes	Male-biased
LRP1B	53 (8%)	41 (14%)	1.84E-03	0.0729	Yes	Male-biased
SPTA1	34 (5%)	30 (10%)	2.05E-03	0.0743	Yes	Male-biased

Table S8. The driver mutations in the TCGA cohort.

Gene	N_nonsilent	N_silent	P value	FDR
KRAS	124	0	0	0
STK11	68	1	0	0
TP53	189	1	4.44E-16	2.79E-12
KEAP1	63	1	8.88E-16	4.19E-12
EGFR	67	1	2.89E-15	1.09E-11
RB1	22	0	5.88E-13	1.85E-09
RBM10	23	1	1.91E-12	5.14E-09
SMARCA4	31	3	9.91E-10	2.34E-06
SMAD4	16	0	4.90E-06	0.010
CDKN2A	17	1	9.03E-06	0.017
ARID1A	24	2	2.65E-05	0.042

Table S9. Comparison of driver and passenger mutations between female and male smokers with LUAD in the TCGA cohort.

Gene	Female	Male	P value	FDR	Driver mutation	Sex-biased
TP53	91 (55%)	67 (49%)	0.248613	0.985129	Yes	Unbiased
KRAS	70 (43%)	46 (33%)	0.098508	0.940898	Yes	Unbiased
STK11	28 (17%)	33 (24%)	0.152132	0.940898	Yes	Unbiased
KEAP1	31 (19%)	29 (21%)	0.666351	1	Yes	Unbiased
EGFR	25 (15%)	7 (5%)	0.004544	0.491729	Yes	Unbiased
RBM10	7 (4%)	15 (11%)	0.04337	0.940898	Yes	Unbiased
SMARCA4	14 (9%)	15 (11%)	0.558542	1	Yes	Unbiased
ARID1A	14 (9%)	7 (5%)	0.264653	0.985129	Yes	Unbiased
RB1	13 (8%)	6 (4%)	0.239669	0.985129	Yes	Unbiased
SMAD4	4 (2%)	6 (4%)	0.520934	0.999522	Yes	Unbiased
CDKN2A	6 (4%)	7 (5%)	0.580685	1	Yes	Unbiased
AMER1	19 (12%)	1 (1%)	8.38E-05	0.081305	No	Female-biased
ZMYM3	15 (9%)	0 (0%)	8.26E-05	0.081305	No	Female-biased

Table S10. Comparison of driver mutations between female and male nonsmokers with LUAD in the TCGA cohort.

Gene	Female	Male	P value	FDR	Driver mutation	Sex-biased
EGFR	21 (51%)	3 (23%)	0.111037	0.589593	Yes	Unbiased
TP53	15 (37%)	5 (38%)	1	1	Yes	Unbiased
KRAS	3 (7%)	3 (23%)	0.142855	0.589593	Yes	Unbiased
STK11	1 (2%)	3 (23%)	0.039339	0.589593	Yes	Unbiased
SMAD4	3 (7%)	2 (15%)	0.583714	1	Yes	Unbiased
KEAP1	1 (2%)	2 (15%)	0.140461	0.589593	Yes	Unbiased
RBM10	0 (0%)	1 (8%)	0.240741	0.589593	Yes	Unbiased
SMARCA4	0 (0%)	1 (8%)	0.240741	0.589593	Yes	Unbiased
CDKN2A	1 (2%)	1 (8%)	0.426974	0.829468	Yes	Unbiased
RB1	2 (5%)	0 (0%)	1	1	Yes	Unbiased
ARID1A	1 (2%)	0 (0%)	1	1	Yes	Unbiased

Table S11. Comparison of driver mutations between female and male smokers with LUAD in the MSKCC-IMPACT cohort.

Gene	Female	Male	P value	FDR	Driver mutation	Sex-biased
STK11	42 (15%)	42 (29%)	0.000739	0.077032	Yes	Male-biased
KEAP1	31 (11%)	34 (23%)	0.000951	0.077032	Yes	Male-biased

Table S12. Comparison of driver mutations between female and male nonsmokers with LUAD in the MSKCC-IMPACT cohort.

Gene	Female	Male	P value	FDR	Driver mutation	Sex-biased
EGFR	41 (66%)	12 (39%)	0.024363	0.087988	Yes	Female-biased
MET	2 (3%)	5 (16%)	0.035195	0.087988	Yes	Male-biased

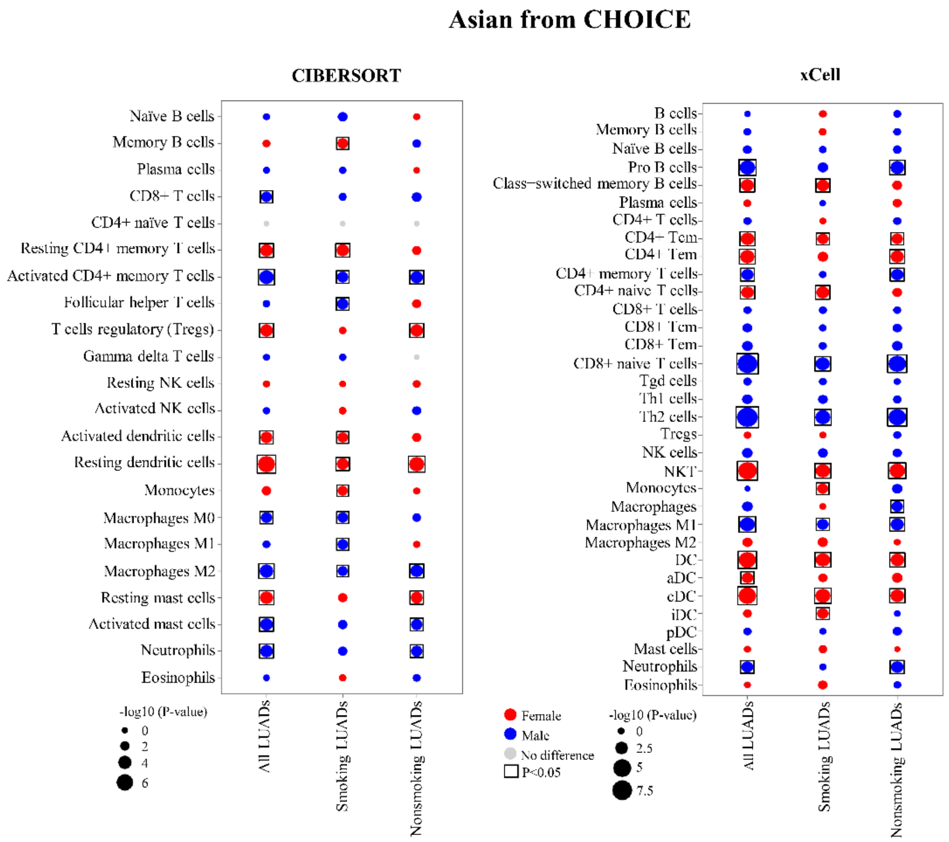

Figure S1. The distributions of immune cell subtypes between male and female LUADs. The bubble plots display the distribution of immune cells between female and male LUADs in the CHOICE cohort. The black boxes represent a statistical significance from Mann-Whitney U test ($P < 0.05$).
